# Supplementary figures and images for: Genetic diversity in migratory bats: Results from RADseq data for three tree bat species at an Ohio windfarm
Source: PeerJ. 2016 Jan 26;4:e1647. doi: 10.7717/peerj.1647 (PMC4730867; doi:10.7717/peerj.1647)

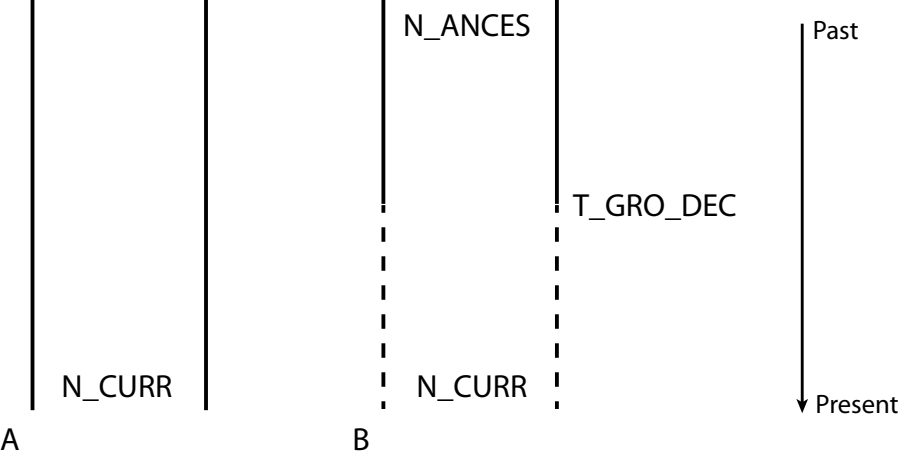

Supplement: Supplemental Information 1 — One model (A) represented a population of constant size, designated by the parameter “N_CURR”. The second model (B) allows for growth or decline (dashed line) from an estimated ancestral population size (“N_ANCES”). This growth or decline was modeled at a constant rate and began at time ‘T_GRO_DEC’. [file peerj-04-1647-s001.pdf]

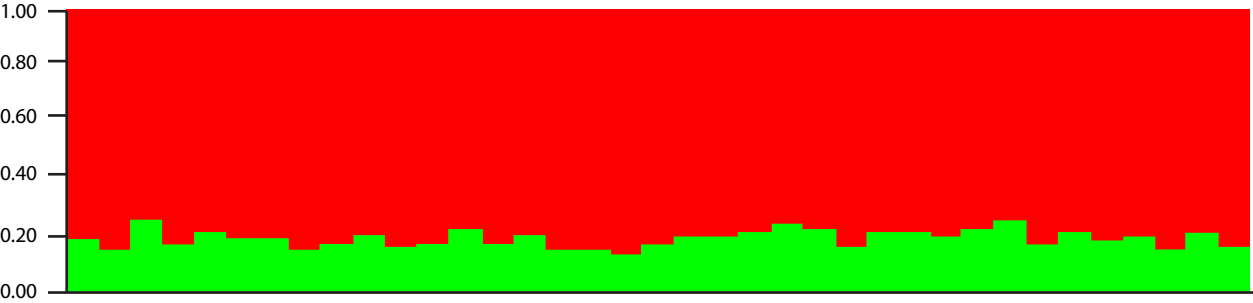

Supplement: Supplemental Information 2 — Although the calculated likelihood was highest at K = 2 for this species, patterns of assignment at K = 2 are not consistent with the presence of genetic structure in this dataset, suggesting that K = 1 may be a better clustering solution in this case. [file peerj-04-1647-s002.pdf]
