# Supplementary material for: Genetic diversity in migratory bats: Results from RADseq data for three tree bat species at an Ohio windfarm
Source: PeerJ. 2016 Jan 26;4:e1647. doi: 10.7717/peerj.1647 (PMC4730867; doi:10.7717/peerj.1647)
Supplement: Supplemental Information 3 [file peerj-04-1647-s003.docx]

**RADseq Library Prep Methods**

RADseq libraries were prepared by first digesting individual samples with 15 units of EcoRI and SbfI restriction enzymes (New England Biolabs) and next ligating unique barcoded adaptors onto each sample. Each individual library was then electrophoresed in a single lane of a 2% low melt point agarose gel with internal 300 and 450 bp standards. Fragments between 300 and 450 bp were isolated using MinElute kits (Qiagen) following the manufacturer’s protocol. Undiluted size-selected products were quantified by qPCR using KAPA library quantification kits (KAPA Biosystems) and the results were used to estimate the number of individual molecules remaining in the library after gel extraction. Samples with less than 1.5x10^5^ molecules were re-prepped to minimize the impact of stochastic sampling effects on locus-specific fragments during library prep, reducing both the amount of missing data in the dataset and associated biases toward homozygous genotype calls. This threshold number of molecules was chosen because samples with fewer molecules tended to show relatively high levels of missing data (M. Sovic, unpublished data).

Each size-selected library meeting the molecule threshold was vacuum centrifuged to 10 uL and PCR amplified in three independent 10 uL PCR reactions. Each reaction included 1X Phusion PCR master mix (New England Biolabs), 5 pmol F and R primer, and 1/3 of the volume of concentrated library. PCR reactions were run for 18 cycles using conditions recommended for the Phusion PCR master mix. Multiple PCR reactions were performed for each library to reduce the occurrence of PCR artifacts. The separate reactions for each individual were then pooled together in a total volume of 30 uL after PCR.

Amplified libraries were purified using AmPure beads. Purified products were quantified with qPCR using KAPA library quantification kits after 1:1500 dilution, and these qPCR results were used to pool individuals in equimolar concentrations for sequencing. Pooled libraries were run in 75-bp runs as partial lanes (ranging from 10% - 40%, depending on the number of individuals in the specific library) on an Illumina HiSeq 2500.
